# An Improved Feature Selection Method Based on Random Forest Algorithm for Wind Turbine Condition Monitoring

**DOI:** 10.3390/s21165654

**Published:** 2021-08-22

**Authors:** Guo Li, Chensheng Wang, Di Zhang, Guang Yang

**Affiliations:** 1 School of Modern Post, Beijing University of Posts and Telecommunications, Beijing 100876, China; liguo@bupt.edu.cn; 2 School of Artificial and Intelligence, Beijing University of Posts and Telecommunications, Beijing 100876, China; yang@bupt.edu.cn; 3 School of Data Science and Media Intelligence, Communication University of China, Beijing 100024, China; dizhang@cuc.edu.cn

**Keywords:** wind turbines, feature selection, FS_RF algorithm, condition monitoring, gated recurrent unit, blade breakages

## Abstract

Feature selection and dimensionality reduction are important for the performance of wind turbine condition monitoring models using supervisory control and data acquisition (SCADA) data. In this paper, an improved random forest algorithm, namely Feature Simplification Random Forest (FS_RF), is proposed, which is capable of identifying features closely correlated with wind turbine working conditions. The Euclidian distances are employed to distinguish the weight of the same feature among different samples, and its importance is measured by means of the random forest algorithm. The selected features are finally verified by a two-layer gated recurrent unit (GRU) neural network facilitating condition monitoring. The experimental results demonstrate the capacity and effectiveness of the proposed method for wind turbine condition monitoring.

## 1. Introduction

Compared with traditional energy sources, wind energy is clean and renewable; thus, wind power has spread worldwide [1,2,3]. However, wind turbines often suffer from frequent malfunctions and failures, which might cause long downtime and significant maintenance costs [4]. For instance, the rotor blades are one of the main components of a wind turbine, and these often fail as the age of the wind farm grows [5]. To prevent high financial losses, condition monitoring and fault prognosis for wind turbines attract a great deal of attention.

In previous studies, condition monitoring methods for wind turbines were mainly carried out with signals collected by sensors. This motivated the research into data-driven wind turbine condition monitoring methods that are capable of estimating working conditions and detecting faults. Nizwan et al. [6] used a Discrete Wavelet Transform (DWT) to analyze vibrational signals in order to achieve fault detection for bearings, where the DWT was employed to decompose signals in different frequency ranges. Sun et al. [7] proposed a method to detect weak features in early faults of rolling bearings in wind turbines. They combined the multiwavelet denoising technique with the threshold of the data-driven block and separated features from noises. Zhang et al. [8] successfully localized the fault planet gear in wind turbine gearboxes using the acoustic emission technique.

Compared with the above monitoring methods, as a comprehensive tool, the supervisory control and data acquisition (SCADA) system has been configured in each wind turbine for working condition supervision. It can provide a large number of parameters that can provide information on the turbine operating condition; therefore, a large number of SCADA data mining methods have been developed. SCADA data were analyzed to construct a model for predicting or detecting the bearing faults of a wind turbine in [9], and faults were predicted by the model 1.5 h before their occurrence. In [10], the authors presented a virtual model to predict two parameters using SCADA data in wind turbines, and the results indicated that the accuracy of the model depended to a large extent on the selected input parameters. The Intelligent System for Predictive Maintenance was applied to monitor the gearbox conditions of wind turbines in [11]. However, among the numerous SCADA parameters, only a few of them are prominent in fault diagnosis and condition monitoring models [12]. Unfortunately, most of the traditional methods [9,10,11,13] have more or less ignored the effects of the interrelation among the SCADA parameters on the model output, to some extent by choosing input parameters according to field experience.

In recent years, deep learning techniques have provided powerful mathematical tools for the fault prognosis and condition monitoring of wind turbines. A large amount of works that have used deep learning techniques were reported in [14]. Jiang et al. [15] employed a denoising autoencoder (DAE) model with time series information from SCADA data to achieve the detection of faults in wind turbines. The optimized long short-term memory (LSTM) neural network, which uses cosine loss, was proposed for the fault diagnosis of a wind turbine gearbox in [16]. Bangalore et al. [17] employed an artificial neural network (ANN) as a condition monitoring method using SCADA data, and the final results proved the effectiveness of the method. All of these approaches [15,16,17] used SCADA data, and input features that are almost manually selected or hand-crafted may restrict the performance of the models. Dimensionality reduction by selecting the most closely related features is a prerequisite to ensure the accuracy of condition estimation models. In [18], the authors developed a prediction model and a diagnosis model using SCADA data for wind turbine generators, where the prediction model was used to predict the remaining useful life of the wind turbine generators. Besides, in [18], the authors also proposed a data preprocessing procedure including data cleaning, feature selection, feature reduction, and data set balancing. In [19], the authors constructed a normal behavior model using support vector regression with a Gaussian kernel to diagnose the faults of wind turbine generators, and the dimensionality of features was reduced by using principal component analysis. Kong et al. [20] introduced a feature selection method with Pearson correlation coefficients in their fault detection model to diagnose the gearbox failures of a wind turbine. Ferreira et al. [21] presented an approach which used decision trees for feature selection and the condition monitoring of tool wear. Wei et al. [22] used a random forest algorithm to select feature parameters and feed them into a constituted deep neural network to detect whether the permanent magnets in a wind turbine had fallen off or not. In [21,22], a method was used that included decision trees to achieve the purpose of condition monitoring. Feature selection was used for variables that can reflect a special component condition in [18,19,20]. However, in this study, the obtained SCADA datasets have only one fault of blade breakages, and no SCADA variables can directly indicate the conditions of blades. Due to this fact, we have to perform feature selection for all SCADA variables.

According to the results of literature studies, better features can simplify the complexity of models and improve the accuracy of condition monitoring models, but the influences of the quality of the selected features on the model performance still remain unclear to date. In this paper, an improved random forest algorithm, the Feature Simplification Random Forest (FS_RF) algorithm, is proposed for the feature selection of SCADA data, in which features that most significantly show the wind turbine’s state are chosen. The gated recurrent unit (GRU) method with the selected features is dedicated to achieving the monitoring of the wind turbine condition. To validate the performance of the proposed method, a comparison is carried out with some other feature selection algorithms. The final results indicate the effectiveness of the proposed method.

This paper is organized as follows. Section 2 presents a brief review of existing feature selection algorithms. The FS_RF algorithm is expounded in Section 3. In Section 4, the experiment setup is depicted and the effectiveness of the proposed feature dimensionality reduction algorithm is evaluated. Finally, the conclusions are given in Section 5.

## 2. Related Feature Selection Algorithms

Generally, there are mainly three families of feature selection algorithms [23,24,25]: filters, wrappers and embedding methods. The differences between these three basic families are in how the learning algorithm is incorporated to evaluate and select features.

In the filter methods [23,26,27,28,29,30], the selected features are evaluated only by the intrinsic properties of the data without running a learning algorithm. These methods neither rely on any machine learning methods nor require cross-validation. For example, the Pearson correlation coefficient method, as one of the filter methods, was introduced by Kong et al. [20] for the feature selection of SCADA data for wind turbine gearbox condition diagnosis. The Pearson correlation coefficient is used to detect the degree of linear correlation between two continuous variables. This method is suitable for solving regression problems but is not appropriate for classification problems. Another filter method is the variance threshold method, which removes only those features whose variance does not satisfy a certain threshold.

The wrapper methods [24,25] select features by “wrapping” the exploration in a learning algorithm and then estimate feature subsets according to the property of the classifier on each candidate feature subset. An obvious drawback of these methods is the high computational cost of the wrapper methods, since the classifier has to be trained and tested for each candidate feature subset. In practice, it is found that using the wrapper methods requires a large amount of computation resources and time when facing high-dimensional SCADA data. Therefore, these methods are not used in this paper.

**Figure 1:**
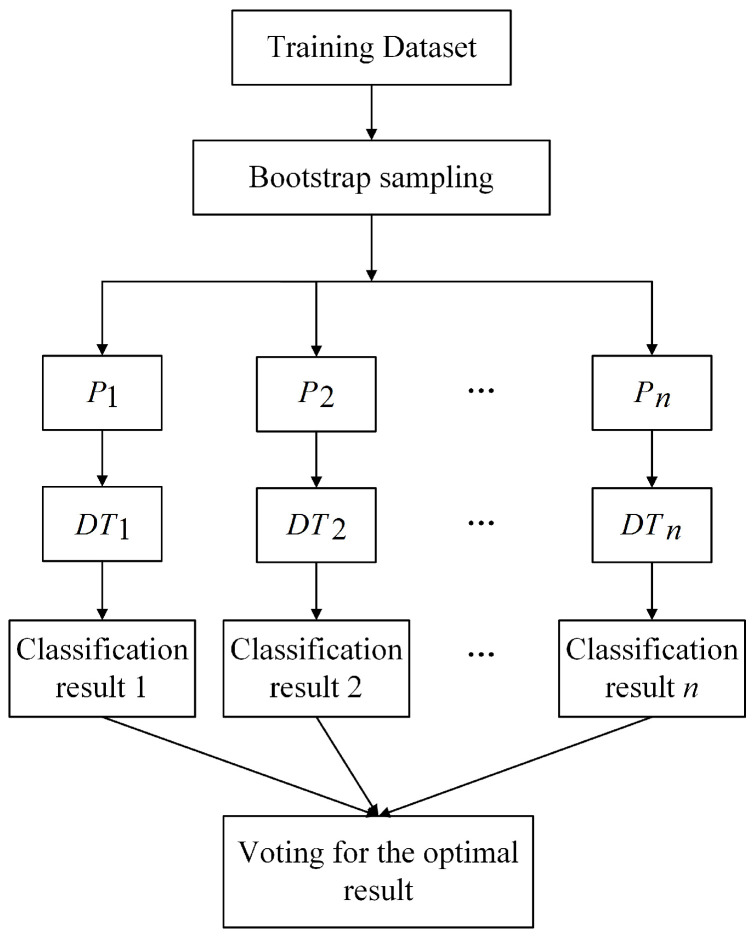
The procedure of the random forest algorithm.

The embedded methods [31,32] integrate the feature search and the learning algorithms into a simple optimization formula, employing the advantages of both the wrapper methods combined with machine learning algorithms and the high computational efficiency of the filter methods. For instance, Wei et al. [22] constructed a deep neural network in order to detect the falling off of permanent magnets from wind turbines and used the random forest algorithm as a feature selection method. The random forest algorithm [33] is an embedded method; it is a combination of tree predictors in which each tree depends on the values of independently sampled random vectors that are identically distributed trees in the forest. The final result is obtained by casting a vote for the most popular class using all the decision trees (DTs). The procedure of the random forest algorithm is summarized below and shown in Figure 1.

Step 1: $P_{1},P_{2},P_{3}...P_{n}$ are sampled randomly from the total training dataset *D* as *n* subsets, and the bootstrap sampling method is used in this process;

Step 2: A DT is constructed for each of the *n* subsets, thus obtaining *n* classification results;

Step 3: Each DT votes for the most popular class, and this can determine the optimal result.

In the DTs, the minimum Gini value is employed as the splitting criterion of the nodes, and the corresponding features are considered to be excellent features. The impurity degree of each node is described by the Gini value [33], calculated using Formula (1):

(1) $$\mathrm{Gini}\left(t\right)=1-\sum_{j=1}^{n}{\left[p\left(j|t\right)\right]}^{2}$$

where p(j∣t) denotes the probability of risk class *j* at node *t*. Once the value of Gini(t) is 0, the sample data at node *t* are recognized as the same risk class. The greater the value of Gini(t), the less available the gained information.

In addition, the L1-SVM algorithm [31], one of the main embedded methods, is an embedded sparse method that uses L1 regularization for linear SVM formulas instead of the standard L2 Margin for selecting features. The L1-SVM algorithm and the random forest random algorithm are employed in this research for comparison.

As depicted above, all the existing algorithms have their own obvious drawbacks while working with SCADA data. Nevertheless, the random forest algorithm behaves better compared with other feature selection methods. However, even in the random forest algorithm, the classification results of the DTs are heavily influenced by the redundant features of the sampled SCADA data. Therefore, this paper proposes the FS_RF algorithm to improve the performance of the random forest algorithm.

## 3. Proposed Algorithm

In [20], to detect whether the permanent magnets in the wind turbines were dislodged, the random forest algorithm was employed for feature selection for construct a condition monitoring model. However, in this paper, in order to diagnose whether the wind turbine blades are broken or not, the feature selection of all SCADA variables is necessary. When facing high-dimensional data, the feature simplification (FS) algorithm can reduce the impact of redundant features on the random forest algorithm.

The FS algorithm removes features that have little impact on the operating state of the wind turbines. The purpose of this process is to decrease the interference of some useless features in the calculation of Gini values in the decision trees of the random forest algorithm. The final results in Section 4.4 indicate that the features that are selected by the FS_RF algorithm are different from features selected using the random forest algorithm.

**Figure 2:**
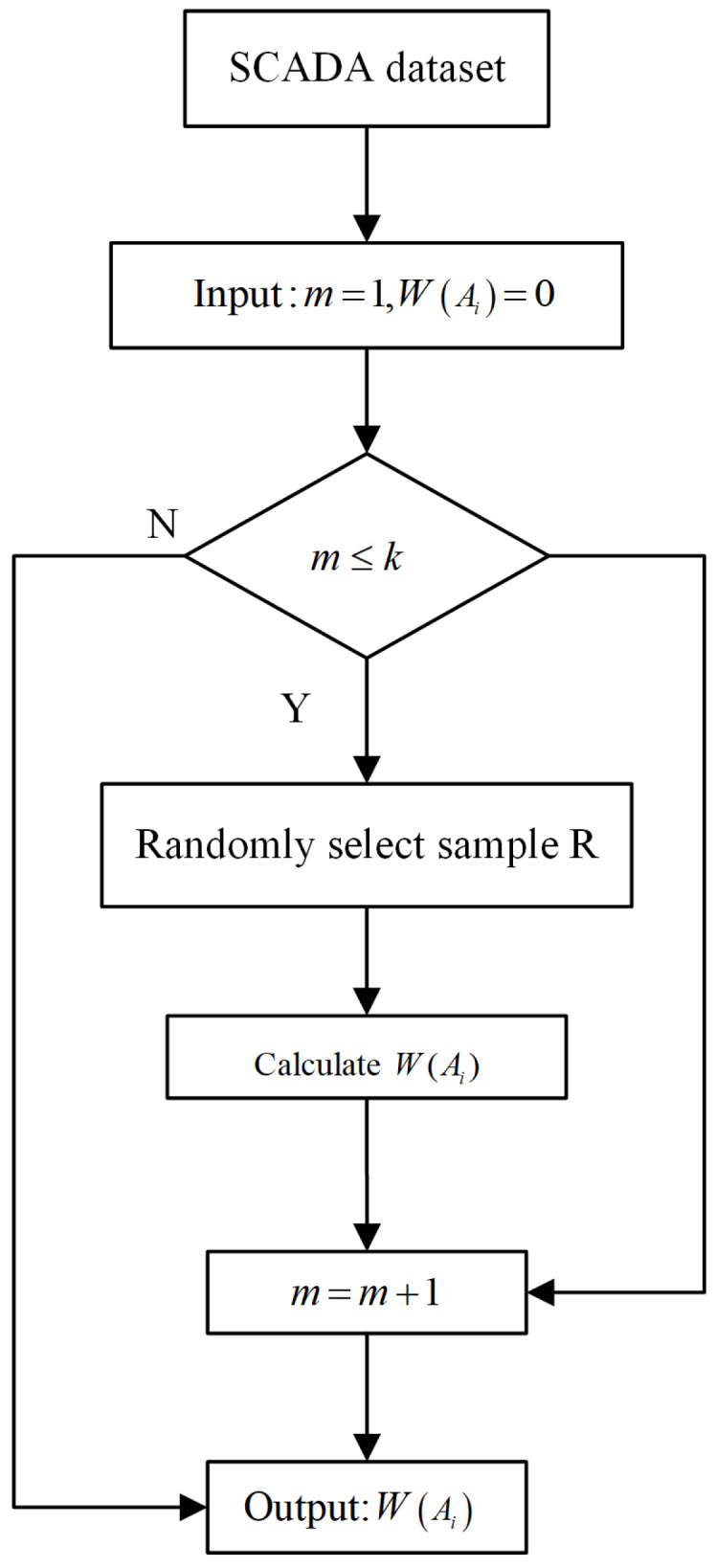
The flowchart of the feature simplification algorithm.

The feature simplification (FS) algorithm mainly calculates the correlation of a feature with the samples from positive and negative classes and assigns corresponding weights to each of the features, where the positive class denotes the SCADA data of the normal working conditions and the negative class the fault. Figure 2 shows the flowchart of the FS algorithm. The detailed algorithm is depicted step by step as follows:

Step 1: Sample *R* is randomly drawn from the SCADA dataset, sample *S* of the same class is adjacent to sample *R*, and sample *D* is drawn randomly from a different class;

Step 2: A feature (denoted as $A_{i}$) is selected from each of the three samples (*R*, *S* and *D*) in turn;

Step 3: The Euclidean distances between feature $A_{i}$ of sample *R* and that of sample *S* are calculated, denoted as $d(R_{A_{i}},S_{A_{i}})$, and those of feature $A_{i}$ between sample *R* and sample *D*, denoted as $d(R_{A_{i}},D_{A_{i}})$;

Step 4: Steps 1 to 3 are repeated, and the weight of each feature $A_{i}$ is computed. The weight computation formula is shown as Formula (2):

(2) $$W\left(A_{i}\right)=W\left(A_{i}\right)-\left(\frac{d\left(R_{A_{i}},S_{A_{i}}\right)}{m}-\frac{d\left(R_{A_{i}},D_{A_{i}}\right)}{m}\right)\left(i=1,2,3,\ldots ,n\right)$$

where $W(A_{i})$ is the weight of feature $A_{i}$ and the initial value of each feature weight is assigned to 0; m denotes the repetitions; and *n* is the total number of features in the SCADA data. In Figure 2, in order to save computation cost, *k* is assigned to 0.6 × *P*, where *P* is the total numbers of samples in a SCADA dataset.

In step 3, if the two Euclidean distances (i.e., $d(R_{A_{i}},R_{S_{i}})$, $d(R_{A_{i}},D_{S_{i}})$) are significantly different, this means that the feature $A_{i}$ has a greater ability to distinguish the positive or the negative samples among *R*, *S*, and *D*, and then the weight of feature $A_{i}$ should be increased. Otherwise, its weight should be decreased.

## 4. A Case Study

In this section, the proposed method is evaluated with 10 min SCADA data on two SCADA datasets collected from two Aeolon58 wind turbines, where the two SCADA datasets contain 418,078 and 404,933 items, respectively. These datasets include normal operating conditions of the wind turbines and failure conditions after the breakage of a blade. A GRU model is constructed to estimate the condition of the wind turbines. The general flow is as follows:

Step 1: The SCADA data are preprocessed by the proposed feature selection method;

Step 2: The SCADA data are divided into training sets and testing sets, and the splitting ratio is set to 0.6;

Step 3: The GRU model is built;

Step 4: The hyper-parameters are initialized (refer to Section 4.4);

Step 5: The training sets are fed to train the GRU model;

Step 6: The correctness of the feature selection method is evaluated.

### 4.1. Data Preprocessing

The SCADA data acquired from a real wind farm supervision system frequently have missing entries, which will lead to discontinuities in the time series. Moreover, a SCADA system usually involves a manifold of variables, which may affect the accuracy of the wind turbine condition estimation model if these missing data are not properly processed [22]. It is therefore necessary to preprocess the SCADA data.

#### 4.1.1. Missing Value Processing

Considering the need to maintain the temporal order of the SCADA data, the missing values of the SCADA data should not be neglected directly. In this paper, the local mean replacement method to pad the missing values is employed, which can be expressed as follows:

(3) $$x_{m}=\frac{\sum_{m-k-1}^{m-1}x_{i}+\sum_{m+k+1}^{m+1}x_{i}}{2k}$$

where $x_{m}$ is the value of the missing data, and *k* represents the number of available data values near the missing data, which is set to 3 in this study, in order to smooth the data series curve.

#### 4.1.2. Feature Selection

In the proposed method, the FS algorithm is incorporated into the feature selection process of the random forest. Firstly, the features with zero or very low weights in the SCADA dataset are initially removed using the feature simplification algorithm; secondly, the random forest algorithm is employed to calculate the importance of features (i.e., columns in the SCADA dataset); and finally, feature selection is achieved based on feature importance.

On the basis of the FS_RF algorithm, the importance of the 75 features in the wind turbine SCADA dataset are computed, and the 28 most important output features are retained as the input of the GRU model. The selected features using the FS_RF algorithm are shown in Table 1.

### 4.2. Constructing the Gated Recurrent Unit Model for Condition Monitoring

When evaluating the conditions of a wind turbine, the data from the SCADA system should be continuously fed into the model in the form of a time series. The common fully-connected neural network is weak in perceiving the change in the conditions of the wind turbine in real time, while the convolutional neural network can only monitor the magnitude of the local change. Obviously, these two methods are both insufficiently sensitive to the input data and cannot reflect the state change of the wind turbine in real time.

A recurrent neural network (RNN) is a type of artificial neural network with directed cycles of dependencies between nodes [34]. This construction allows the network to retain previous state information between successive time steps. The value of every time step is considered, affecting the temporal result. Generally, given a $sequenceX=[x_{1},x_{2},\ldots ,x_{n}]$, $x_{t}\in R^{k}$ is the input of time step *t*. The process of the RNN preserving states can be defined by the following state transfer function [35,36]:

(4) $$h_{t}=f\left(Wx_{t}+Uh_{t-1}+b\right)$$

where $U\in R^{d\times d}$ represents the matrix between the adjacent time-step of hidden layers and itself; $W\in R^{d\times k}$ is the matrix, which denotes the ordinary weights between the input layer and hidden layers; $b\in R^{d}$ is the bias parameter; *U*, *W* and *b* are shared parameters and can learn during model training; $h_{t}\in R^{d}$ denotes the corresponding hidden state when the input is $x_{t}$; and *f*, which is widely used in RNNs, is the hyperbolic tangent activation function.

Nonetheless, standard RNNs suffer from gradient disappearance and gradient explosion during training, which may make it difficult to obtain satisfactory results. More importantly, standard RNNs are unable to remember long-term data and discard relevant prior states—a problem known as “fading memory” [37].

**Figure 3:**
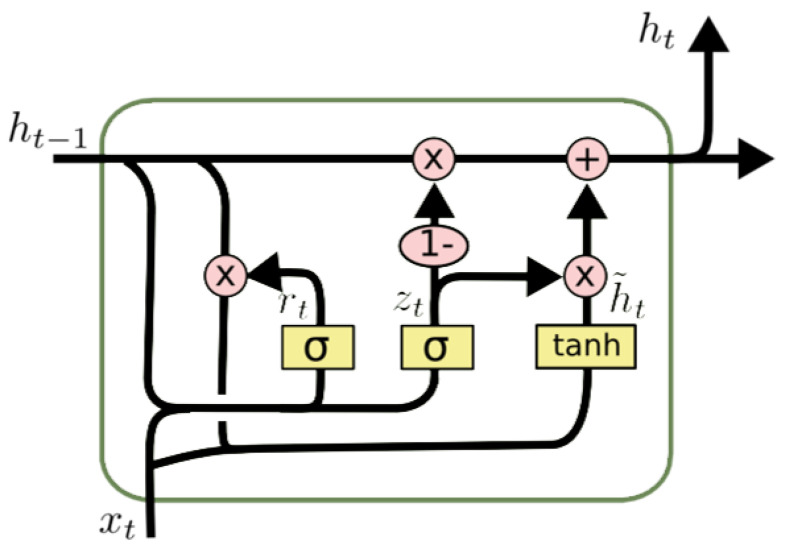
The structure of GRU.

The gated recurrent unit (GRU) evolved on the basis of the RNN and has become popular due to its better information storage and ability to access prior conditions. In contrast to traditional RNNs, the hidden unit of the GRU is replaced by a gated unit, which does not require a separate storage unit to regulate the flow of information within the unit. The structure of this model is shown in Figure 3.

The calculation of the GRU layer can be described as follows [34]:

(5) $$z_{t}=\sigma_{g}\left(W_{z}x_{t}+U_{z}h_{t-1}+b_{z}\right)$$

(6) $$r_{t}=\sigma_{g}\left(W_{r}x_{t}+U_{r}h_{t-1}+b_{r}\right)$$

(7) $${\tilde{h}}_{t}=\tanh\left(W_{h}x_{t}+U_{t}\left(r_{t}⊙h_{t-1}\right)\right)$$

(8) $$h_{t}=\left(1-z_{t}\right)⊙h_{t-1}+z_{t}⊙{\tilde{h}}_{t}$$

where all $W\in R^{d\times k}$, $U\in R^{d\times d}$ and $b\in R^{d}$, as the included learnable parameters, should be shared by each step, and they are all able to learn in the period of the GRU model training; ⊙ represents the element-wise product; *d* and *k* are hyper-parameters representing the dimensions of the input and hidden vector; $h_{t}$ is the output vector, which includes information of the current unit when the input vector is $x_{t}$; $z_{t}$ and $r_{t}$ are the vectors for update gate and reset gate; $\sigma_{g}$ is the sigmoid activation function; and tanh is the hyperbolic tangent activation function.

The GRU model can resolve the drawbacks of the standard RNNs. The update gate vector and the reset gate vector are employed to decide whether the information should be remembered or forgotten and to learn adaptive weights of different features. Moreover, compared with the long short-term memory (LSTM) network, the GRU has better performance with fewer parameters to suppress overfitting [38].

### 4.3. Loss Function and Evaluation Criteria

The evaluation of the feature selection algorithm is performed according to the accuracy of the model. We use binary cross-entropy as a loss function, and its mathematical expression could be expressed as follows:

(9) $$l_{n}=-\left(y_{n}*\log\left({\hat{y}}_{n}\right)+\left(1-y_{n}\right)*\log\left(1-{\hat{y}}_{n}\right)\right)$$

(10) $$\mathrm{loss}\left(z,y\right)=\mathrm{mean}\left\{l_{0},l_{1},l_{2},\cdots ,l_{N-1}\right\}$$

where *N* denotes the total number of samples; ${\hat{y}}_{n}$ is the probability that the *n*th sample is a positive case; and $y_{n}$ the true label of the *n*th sample.

To measure the influence of the feature selection on the deep learning model, the *F*1-Score is used as a criterion. Here, “0” denotes a healthy condition of the wind turbine and “1” represents an abnormal condition. The basic parameters in the standard performance metrics are adopted: true positive (TP), false positive (FP), false negative (FN), and true negative (TN).

As a statistical measurement, the *F*1-Score combines the precision and recall of the classification model, with a maximum value of 1 and a minimum value of 0. The *F*1-Score can be mathematically expressed as follows:

(11) $$\;\mathrm{Accuracy}\;=\frac{TP+TN}{TP+FP+FN+TN}$$

(12) $$\;\mathrm{Precision}\;=\frac{TP}{TP+FP}$$

(13) $$\;\mathrm{Recall}\;=\frac{TP}{TP+FN}$$

(14) $$F1-\;\mathrm{Score}\;=\frac{2TP}{2TP+FN+FP}=\frac{2\cdot \;\mathrm{Precision}\;\cdot \;\mathrm{Recall}\;}{\;\mathrm{Precision}\;+\;\mathrm{Recall}\;}$$

The aforementioned metrics are specific to each category, and they measure the capacity of the condition monitoring model to distinguish certain circumstances (i.e., fault) from normal circumstances (i.e., health). In addition, accuracy is also employed to evaluate the overall model performance for the positive class and negative class.

### 4.4. Experiment Results

In this section, a GRU model is constructed and applied to the wind turbine condition estimation. Two SCADA datasets are employed to evaluate the proposed algorithm, in which both healthy and failure operating condition data are recorded. In the proposed method, the selected features, preprocessed using the FS_RF algorithm, are fed into the GRU model. The detailed setup of the GRU model is shown in Table 2.

To further evaluate the performance of the FS_RF algorithm, a comparison is conducted with other feature selection algorithms—i.e., the random forest algorithm, the variance threshold algorithm, and the L1-SVM algorithm—where the threshold of the variance threshold method is set to 0.4. The results of different feature selection methods are described in Table 3 and Table 4, respectively.

The experimental results show that different feature selection methods result in a variance in the number of selected features. In Table 3, the *F*1-Score of the GRU condition estimation model which uses the proposed method reaches 95.21, surpassing the method in second place by about 0.41. In Table 4, the *F*1-Score of the best performing model using the FS_RF algorithm is 91.24, which exceeds the model in second place by approximately 0.97.

As shown in Table 3 and Table 4, the FS_RF algorithm performs well on both data samples. Further, 28 features with a higher impact on the metrics are selected using the FS_RF algorithm, while the random forest algorithm selects only 22 features. This means that the final *F*1-Score of the model using the FS_RF algorithm is higher than that of the random forest algorithm.

### 4.5. Discussion

In this study, we propose a FS algorithm to optimize the random forest algorithm for SCADA data. The FS_RF algorithm behaves more efficiently when choosing the important features from the data sample than other algorithms. The reason for this may be that the features selected by the FS algorithm reduce the complexity of the later calculation of feature importance using the random forest algorithm. In addition, it is worth noting that none of the condition monitoring models constructed for sample 2 using the four feature selection methods performed as well as those for sample 1. This may have been caused by the degree of the failures of the wind turbine; i.e., minor failures could cause insignificant variations in the parameters of the SCADA data, while severe faults cause remarkable changes, which in turn affects the diagnosis ability of the GRU models.

## 5. Conclusions

Feature selection and dimensionality reduction on the SCADA data of wind turbines remain a perplexing problem. Although prior research has provided hope for a better result of condition estimation, there still remains room for improvement.

This paper proposes the FS_RF algorithm for feature selection and dimensionality reduction on SCADA data, and the approach is evaluated using a GRU deep learning model. The proposed algorithm is able to select features from SCADA datasets that better denote the operating state of the wind turbines by means of computing the weight of a feature using Euclidian distances among sample datasets and measuring its importance. The results obtained in the experiments demonstrate the applicability of the proposed method.

In addition, the findings of this research have a number of important implications for future practice by providing a reference for feature selection and dimensionality reduction on massive datasets in other fields.

## Figures and Tables

**Table 1:** Selected SCADA features.

No	Feature	No	Feature
1	Nacelle temperature	2	Hub temperature
3	Reactive power control status	4	Generator active power
5	Converter grid side voltage	6	Blade 1 battery box temperature
7	Blade 2 battery box temperature	8	Blade 3 battery box temperature
9	Blade 1 converter box temperature	10	Blade 2 converter box temperature
11	Blade 3 converter box temperature	12	Blade 1 angle
13	Blade 2 angle	14	Blade 3 angle
15	Main bearing temperature 1	16	Main bearing temperature 2
17	Engine room control cabinet temperature	18	Hub control cabinet temperature
19	Generator stator temperature 1	20	Generator stator temperature 2
21	Generator stator temperature 3	22	Generator stator temperature 4
23	Generator stator temperature 5	24	Generator stator temperature 6
25	Wind measurement tower temperature	26	Converter inlet pressure
27	Converter outlet pressure	28	Absolute wind direction

**Table 2:** Structure and hyper-parameter setup of the GRU model.

	Structure and Hyper-Parameter Setting	Values
Structure	Input size	28
	The number of neurons in the GRU layer	50
	The number of neurons in the fully connected layer	10
	The number of neurons in the output layer	2
Training settings	Batch size	64
	Learning rate	0.01
	The number of GRU layers	2
	Dropout rate in the GRU layers	0.2
	The total number of epochs	50

**Table 3:** Results on the sample 1.

Method	Num of Features	Accuracy	Precision	Recall	*F*1-Score
VarianceThrehold-GPU	57	90.31	91.57	89.31	91.79
L1-SVM-GPU	61	93.18	94.32	92.34	94.17
RandomForest-GRU	22	94.00	94.71	93.37	94.80
FS_RF-GRU(Proposed)	28	94.50	95.13	93.93	95.21

**Table 4:** Results on the sample 2.

Method	Num of Features	Accuracy	Precision	Recall	*F*1-Score
VarianceThrehold-GPU	57	81.65	81.59	81.75	82.44
L1-SVM-GPU	61	89.83	89.81	89.92	90.27
RandomForest-GRU	22	88.64	88.60	88.78	88.94
FS_RF-GRU(Proposed)	28	90.77	90.71	90.83	91.24

## Data Availability

The data presented in this study are available on request from the corresponding author.

## Abbreviations

The following abbreviations are used in this manuscript:

MDPI	Multidisciplinary Digital Publishing Institute
DOAJ	Directory of open access journals
TLA	Three letter acronym
LD	Linear dichroism
